# Conflicts of Interest among Authors of Clinical Practice Guidelines for Glycemic Control in Type 2 Diabetes Mellitus

**DOI:** 10.1371/journal.pone.0075284

**Published:** 2013-10-14

**Authors:** Susan L. Norris, Haley K. Holmer, Lauren A. Ogden, Brittany U. Burda, Rongwei Fu

**Affiliations:** 1 Department of Medical Informatics and Clinical Epidemiology, Oregon Health & Science University, Portland, Oregon, United States of America; 2 Kaiser Permanente Center for Health Research, Portland, Oregon, United States of America; 3 Department of Public Health and Preventive Medicine, Department of Medical Informatics and Clinical Epidemiology, Oregon Health & Science University, Portland, Oregon, United States of America; York University, Canada

## Abstract

**Background:**

Conflict of interest (COI) is an important potential source of bias in the development of clinical practice guidelines (CPGs).

**Objectives:**

To examine rates of disclosure of COI, including financial interests in companies that manufacture drugs that are recommended in CPGs on glycemic control in type 2 diabetes mellitus, and to explore the relationship between recommendations for specific drugs in a guideline and author COI.

**Methods:**

We identified a cohort of relevant guidelines from the National Guideline Clearinghouse (NGC) and abstracted COI disclosures from all guideline authors for this observational, cross-sectional study. We determined which hypoglycemic drugs were recommended in each guideline, and explored the relationship between specific disclosures and whether a drug was recommended.

**Results:**

Among 13 included guidelines, the percentage of authors with one or more financial disclosures varied from 0 to 94% (mean 44.2%), and was particularly high for two US-based guidelines (91% and 94%). Three guidelines disclosed no author financial COI. The percentage of authors with disclosures of financial interests in manufacturers of recommended drugs was also high (mean 30%). On average, 56% of manufacturers of patented drugs recommended in each guideline had one or more authors with a financial interest in their company. We did not find a significant relationship between financial interests and whether a drug was recommended in our sample; US-based guidelines were more likely to make recommendations for a specific drug compared to non-US based guidelines.

**Discussion:**

Authors of this cohort of guidelines have financial interests directly related to the drugs that they are recommending. Although we did not find an association between author COI and drugs recommended in these guidelines and we cannot draw conclusions about the validity of the recommendations, the credibility of many of these guidelines is in doubt.

## Introduction

High quality clinical practice guidelines (CPGs) are an important tool used to optimize patient care: they provide recommendations informed by a systematic review of evidence and an assessment of the benefits and harms of alternative care options [1]. CPGs set the standards for medical care [2] and have the potential to influence the care delivered by a large number of healthcare providers and patients [3]. The quality of CPGs is therefore critically important: high-quality, or trustworthy guidelines promote the use of effective clinical services, decrease undesirable practice variation, reduce the use of services that are of minimal or questionable value, increase the use of effective but underused services, and target services to populations most likely to benefit [4].

Conflict of Interest (COI) is an important potential source of bias in the development of CPGs. A COI is a set of conditions in which professional judgment concerning a primary interest (such as the health and wellbeing of a patient or the validity of research), is unduly influenced by a secondary interest [5]. Physician-industry relationships [6] and industry funding of research [7,8] are frequent and industry funding is increasing in prevalence. There are data suggesting an association between author or funder COI and study outcomes [9-14], between industry relationships and physician behavior [15] or expressed opinions [16,17], and between COI and conclusions in systematic reviews [18]. 

Data on disclosures of industry relationships of CPG sponsors and authors suggest that the prevalence is high [19-26] and disclosure rates are suboptimal. In a random sample of 250 CPGs in the National Guideline Clearinghouse [25], only 60% of CPGs indicated that they had collected disclosures from guideline authors, and of CPGs where there were disclosures for all authors, 60% had one or more authors with a COI. 

The objectives of this study were to examine the prevalence of COI among the authors of CPGs on glycemic control in type 2 diabetes mellitus (diabetes); to determine if CPG authors disclosed payments from companies that manufacture drugs specifically mentioned in the guideline; and to explore whether financial interests of authors were correlated with specific drugs recommended in a guideline. We focused on diabetes mellitus guidelines because of the high and increasing burden of disease, with 347 million people affected worldwide, of which over 90% have type 2 diabetes [27]. In addition, there are a large number of clinical practice guidelines and a vast array of pharmacotherapeutic agents used for treatment of this disease. 

## Methods

 We identified CPGs listed in the National Guideline Clearinghouse (NGC )[28] on either of the dates of our search (February 15, 2012 and June 6, 2012) that provided guidance on drugs for glycemic control in type 2 diabetes, including adults, children, and pregnant women, in any setting or geographic location. We searched on two separate dates in order to capture a broad cohort of related CPGs which are continually updated and archived in NGC. We excluded guidelines that did not provide COI disclosures. 

The NGC is an initiative of, and is funded by, the US Agency for Healthcare Research and Quality (AHRQ) of the U.S. Department of Health and Human Services. The mission of the NGC is to provide health professionals, delivery systems, and payers access to objective and detailed information on clinical practice guidelines and to disseminate and implement high quality guidelines [28]. The inclusion criteria for guidelines within the NGC are: 1) The clinical practice guideline contains systematically developed statements that include recommendations, strategies, or information that assists physicians and/or other health care practitioners and patients to make decisions about appropriate health care for specific clinical circumstances. 2) The clinical practice guideline was produced under the auspices of medical specialty associations; relevant professional societies, public or private organizations, government agencies at the Federal, State, or local level; or health care organizations or plans. 3) Corroborating documentation can be produced and verified that a systematic literature search and review of existing scientific evidence published in peer reviewed journals was performed during the guideline development. 4) The full text guideline is available upon request in the English language. 5) The guideline was developed, reviewed, or revised within the last 5 years. 

Included guidelines addressed pharmacotherapy for glycemic control such as treatment algorithms or recommendations on specific hypoglycemic agents (including insulin) or general classes of such agents. The full text of the CPGs was available in the public domain, either published in the peer-reviewed literature, retrieved from the sponsor’s web-site, or purchased from the CPG developer or sponsor. 

We abstracted COI disclosure statements (both financial and nonfinancial interests) for each guideline author from the CPG itself and from the summary in the NGC. For nonfinancial interests, we included any disclosure that was not reported in, or referable to, monetary units. For each CPG we searched the sponsor website for a COI policy directly relevant to guideline development. 

 In order to evaluate the relationship between the financial conflicts disclosed by guideline authors and drug recommendations in the guideline, we identified all hypoglycemic agents available in the U.S. using Epocrates Online (Epocrates, Inc., San Mateo, CA, 2012), including oral agents, and injectable agents including insulin. (Vildagliptin was the only brand-name (on patent) drug recommended in our international guideline cohort which is not currently approved for use in the US.) Two coauthors (SLN, BUB), blinded to the organization producing the guideline and to author COI disclosures, independently determined what drugs (both on-patent and available generically) were specifically recommended in each guideline, and came to consensus when disagreements occurred. Because most hypoglycemic agents can be used in a wide variety of clinical scenarios, in order to make a reproducible determination for each drug, we defined a drug as “recommended” when it was suitable for use in any patient population, even if there were significant restrictions suggested for its usage. If a drug was mentioned only in the evidence review (either in a separate document or in the guideline itself) and not in the guidance portion of the document, this was not considered a “recommended” drug. If a drug class was recommended but there was no mention of a specific drug, drugs within that class were not considered to have been specifically “recommended.” 

### Statistical Analysis

Since this was an observational, exploratory study, we did not perform sample size calculations. Kappa statistic was calculated to examine the agreement between the two assessments of whether a drug was recommended in each guideline. Guideline characteristics were summarized using descriptive statistics. A logistic regression model with generalized estimating equation (GEE) was used to examine the relationship between whether a drug was recommended (Yes vs. No), and the financial interests disclosed by the authors as well as characteristics of the CPG. The recommendation for all drugs was assessed in a single model and the GEE approach took into account the correlation among recommendations within each CPG. Variables assessed in the logistic regression model included the percent of authors with COI for each recommended drug, percent of authors with a disclosed interest in any drug manufacturer, whether the chair of the CPG had a COI for each recommended drug, and whether the chair had a disclosed interest in any drug manufacturer. Characteristics of the GPGs that were examined in the regression model included the number of authors for each CPG, year of publication, country of the CPG (US vs. non-US), type of organization (government vs. non-government, professional society, academic institution, non-profit organizations), and whether the CPG developer had a COI policy. All drugs evaluated in the included guidelines also entered the model as multiple dummy variables to control for the differences in recommendations among drugs. 

## Results

Thirteen guidelines fulfilled our inclusion criteria (Table **1**) [29-41] including six from the US and three from Europe. The majority were from government agencies (five) or medical specialty societies (four). There was a wide range of number of authors (5 to 27) and number of manufacturers with patented drugs recommended (0 to 11) across guidelines. Nine guideline developers had an accessible COI policy, of which five made reference to nonfinancial interests [29,32,36,37,39]. No guideline was funded by industry because of the criteria for inclusion in the NGC[28]. 

**Table 1:** Characteristics of included guidelines and the prevalence of conflicts of interest among guideline authors.

Developer (funder, if different); Country	Date released	Organization type	Population; Guideline focus	No. of authors	No. of manufacturers with patented drugs recommended in the CPG	No. of authors with any financial COI (% of total no. of authors)	No. of authors with relevant financial COI* (% of total no. of authors)	Chair with any financial COI (Y/N)	Chair with relevant financial COI* (Y/N)	Manufacturers of patented drugs recommended in the CPG with which the chair has a COI (no., %)	Manufacturers of patented drugs recommended in the CPG with which 1 or more authors has a COI (no., %)
AACE[31]; USA	March 2011	Medical Specialty Society	Adults and children; Diabetes comprehensive care plan	23	5	21 (91)	19 (83)	Y (4 of 4 co-chairs)	Y (3 of 4 co-chairs)	0,3,3,5 (4 chairs); 0, 60,60,100	5 (100)
ACP[30]; USA	February 2012	Medical Specialty Society	Adults; Oral pharmacologic treatment of DM2	14	7	2 (14)	2 (14)	N	N	0 (0)	2 (29)
ADA[29]; USA	January 2012	Medical Specialty Society	Adults, children, pregnant women; Standards for medical care for DM1, DM2, and diabetes in pregnancy	16	11	15 (94)	10 (63)	Y	Y	5 (45)	8 (73)
CADTH[32] (Health Canada); Canada	May 2009	Nonprofit Organization	Adults, children, pregnant women; Use of insulin analogues in DM1, DM2, and diabetes in pregnancy	12	3	7 (58)	5 (42)	Y	Y	1 (33)	3 (100)
CADTH[33] (Health Canada); Canada	August 2010	Nonprofit Organization	Adults; DM2 inadequately controlled with metformin	12	8	6 (50)	5 (42)	N	N	0 (0)	6 (75)
ESC[34]; Europe	May 2012	Medical Specialty Society	Patients with established atherosclerotic CVD and asymptomatic individuals at increased risk for CVD; CVD prevention	27	0	20 (74)	0 (0)	Y	N	0 (0)	NA
ICSI[35] (Funded by members' dues and sponsoring health plans); USA	July 2010	Nonprofit Organization	Adults; Diagnosis and management of prediabetes and DM2	13	11	7 (54)	4 (31)	Y (2 of 2 co-chairs)	N (0 of 2 co-chairs)	0 (0)	5 (45)
NCC - WCH[36]: UK	March 2008	Government Agency [Non-U.S.]	Women with diabetes who are planning to become pregnant, who are already pregnant, and their newborn babies; Diabetes in pregnancy	12	2	6 (50)	4 (33)	N	N	0 (0)	2 (100)
NICE[37]; UK	April 2010	Government Agency [Non-U.S.]	Adults; Liraglutide in DM2	27	1	0 (0)	0 (0)	N	N	0 (0)	0 (0)
QPHC[38]; Saudi Arabia	2011	Government Agency [Non-U.S.]	Adults (non-pregnant) ; individuals at increased risk for CV D and DM2; Cardiometabolic risk management in primary care	12	7	0 (0)	0 (0)	N	N	0 (0)	0 (0)
SIGN[39]; Scotland	March 2010	Government Agency [Non-U.S.]	Adults and children; Management of DM1, DM2, GDM	11	8	7 (64)	6 (55)	Y	Y	3 (38)	6 (75)
UMHS[40]; USA	December 2009	Academic Institution	Adults; Management of DM2 in primary care	8	9	2 (25)	2 (25)	N	N	0 (0)	6 (67)
WDPCP[41]; USA	March 2011	Government Agency	Adults, pregnant woman; Management of glycemic control in DM1, DM2, GDM	5	11	0 (0)	0 (0)	NA (no chair)	NA (no chair)	NA	0
**Summary**: US: 6; Europe: 3; Other: 3	2008: 1; 2009: 2; 2010: 4; 2011: 3; 2012: 3	Government agency: 5; Medical specialty society: 4; Nonprofit organization: 3; Academic institution: 1		Range: 5-27 Mean: 14.8 Median: 12	Range: 0-11 Mean: 6.4 Median: 7	Range: 0-94% Mean: 44.2% Median: 50%	Range: 0-83% Mean: 29.9% Median: 31%	Y: 6; N: 6**	Y: 4; N: 8**	Range: 0-100%; Mean: 18%; Median: 0%	Range: 0-100%; Mean: 56.1%; Median: 70%

(*) Relevant COI refers to a financial interest in one or more of the manufacturers of patented drugs recommended in the CPG.

(**) One guideline had no designated chair.

CPG, clinical practice guideline; COI, conflict of interest; CVD, cardiovascular disease; DM 1, type 1 diabetes mellitus; DM2, type 2 diabetes mellitus; GDM, gestational diabetes mellitus; N, no; NA, not applicable; No., number; Y, yes

**Abbreviations for guideline developers:** American Association of Clinical Endocrinologists, AACE; American College of Physicians, ACP1; American College of Physicians, ACP2; American Diabetes Association, ADA; American Medical Directors Association, AMDA; Canadian Agency for Drugs and Technologies in Health, CADTH; European Society of Cardiology, ESC; Institute for Clinical Systems Improvement, ICSI; International Diabetes Center, IDC; International Diabetes Federation, IDF; Joslin Diabetes Center, JDC; National Kidney Foundation, KDOQI; National Collaborating Centre for Women's and Children's Health, NCC - WCH; National Collaborating Centre for Acute and Chronic Conditions, NCC-ACC; National Health Care for the Homeless Council, NHCHC; National Institute for Health and Clinical Excellence, NICE; New York State Department of Health, NY DoH; Qatif Primary Health Care, QPHC; Scottish Intercollegiate Guidelines Network, SIGN; University of Michigan Health System, UMHS; Department of Veterans Affairs/Department of Defense, VA/DoD; Wisconsin Diabetes Prevention and Control Program, WDPCP

The percentage of authors with one or more financial COI varied across guidelines from 0 to 94% (mean 44.2%, median 50.0%), and was particularly high for two US-based groups (94% for the American Diabetes Association (ADA )[29] and 91% for the American Association of Clinical Endocrinologists (AACE) [31]). On the other hand, three guidelines disclosed no author financial COI (National Institute for Clinical Excellence (NICE) [37], Qatif Primary Health Care (QPHC) [38], and Wisconsin Diabetes Prevention and Control Program (WDPCP) [41]).

**Figure 1:**
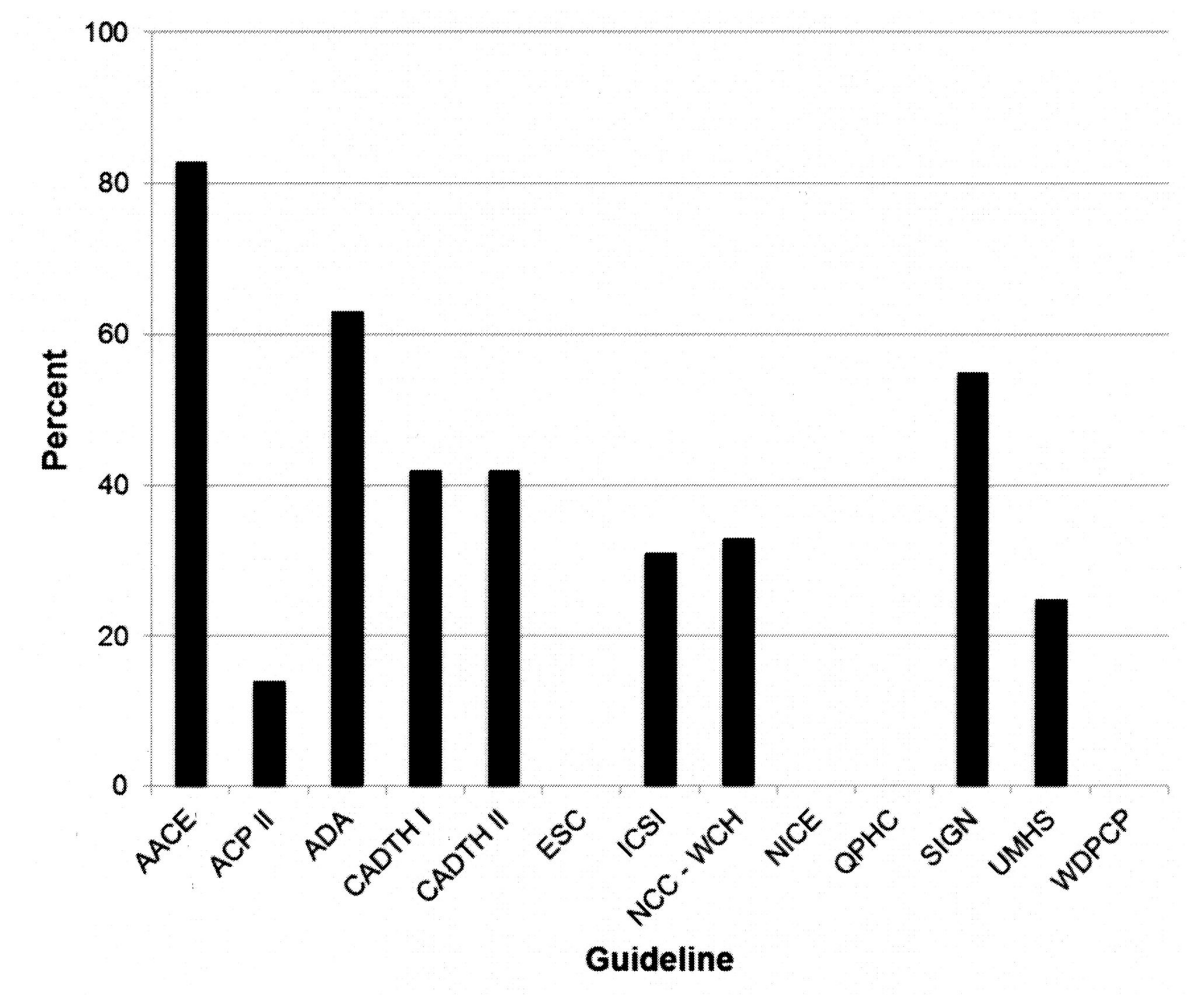
Guideline authors with financial interests in recommended drugs. Percentage of authors of clinical practice guidelines with a financial interest in one or more of the manufacturers of patented drugs recommended in each guideline. Abbreviations. See list for Table 1.

**Figure 2:**
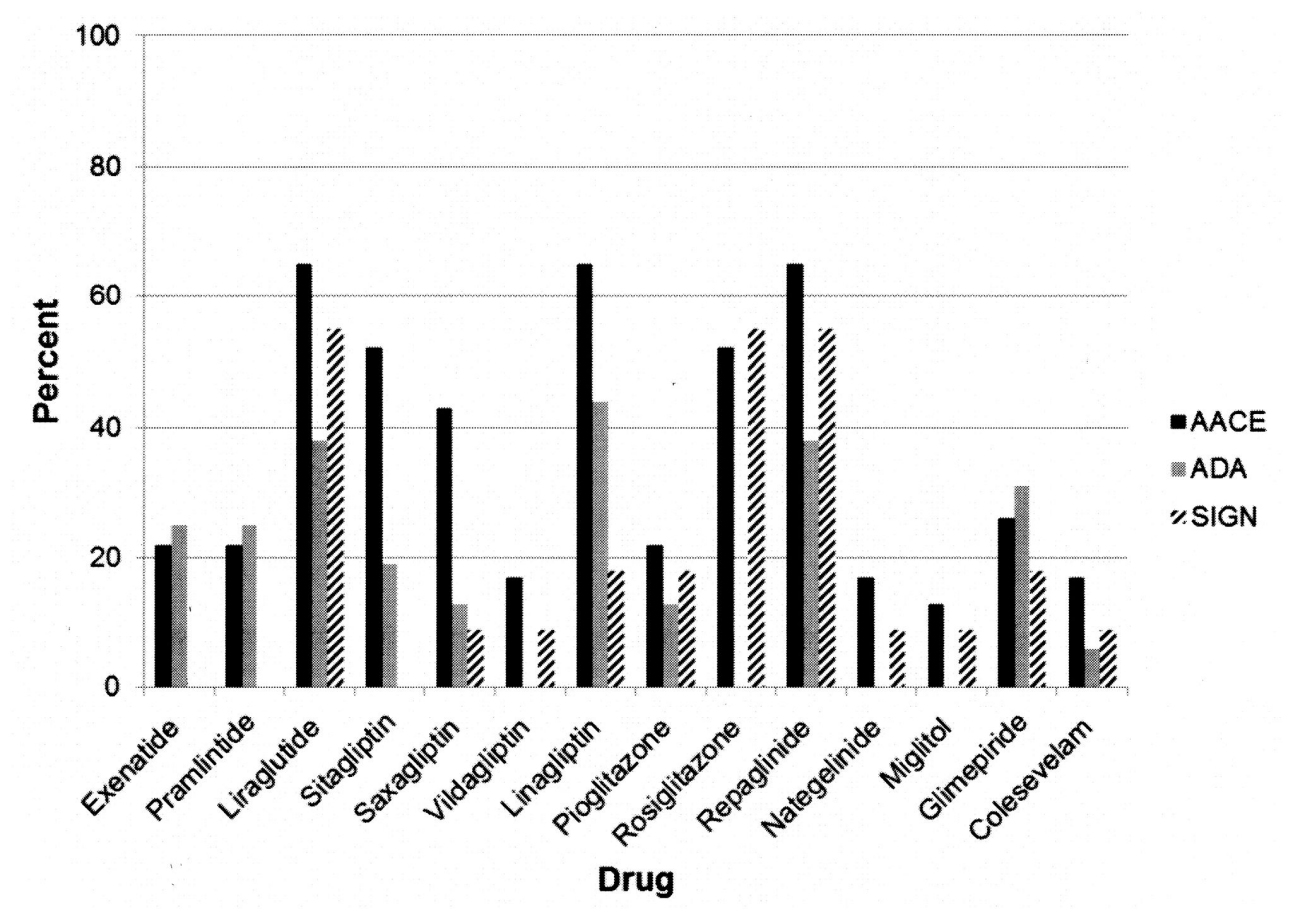
Financial interests in specific drugs among three selected guidelines. Percent of authors of three clinical practice guidelines with disclosures of financial interests in the manufacturers of specific drugs. Abbreviations. See list for Table 1.

The percentage of authors with relevant COI (i.e., financial interests disclosed for companies manufacturing patented drugs recommended in the guideline) was also high for the two American organizations (AACE 83% [31], ADA 63% [29]), with a mean of 30% (median 31%) across all guideline groups (Figure **1**, Table **1**, Table **S1**). In three guidelines [29,31,39,42] more than 50% of authors had a financial interest in patented drugs recommended in the guideline; the percentage of authors with financial interests varied considerably among the various drugs and across the three guidelines (Figure **2**). In addition to the three guidelines with no disclosed author COI [37,38,41], one guideline [34] contained no recommendations for patented drugs (only for metformin), and therefore no relevant COI. No guideline authors disclosed any interests that were not financial.

**Figure 3:**
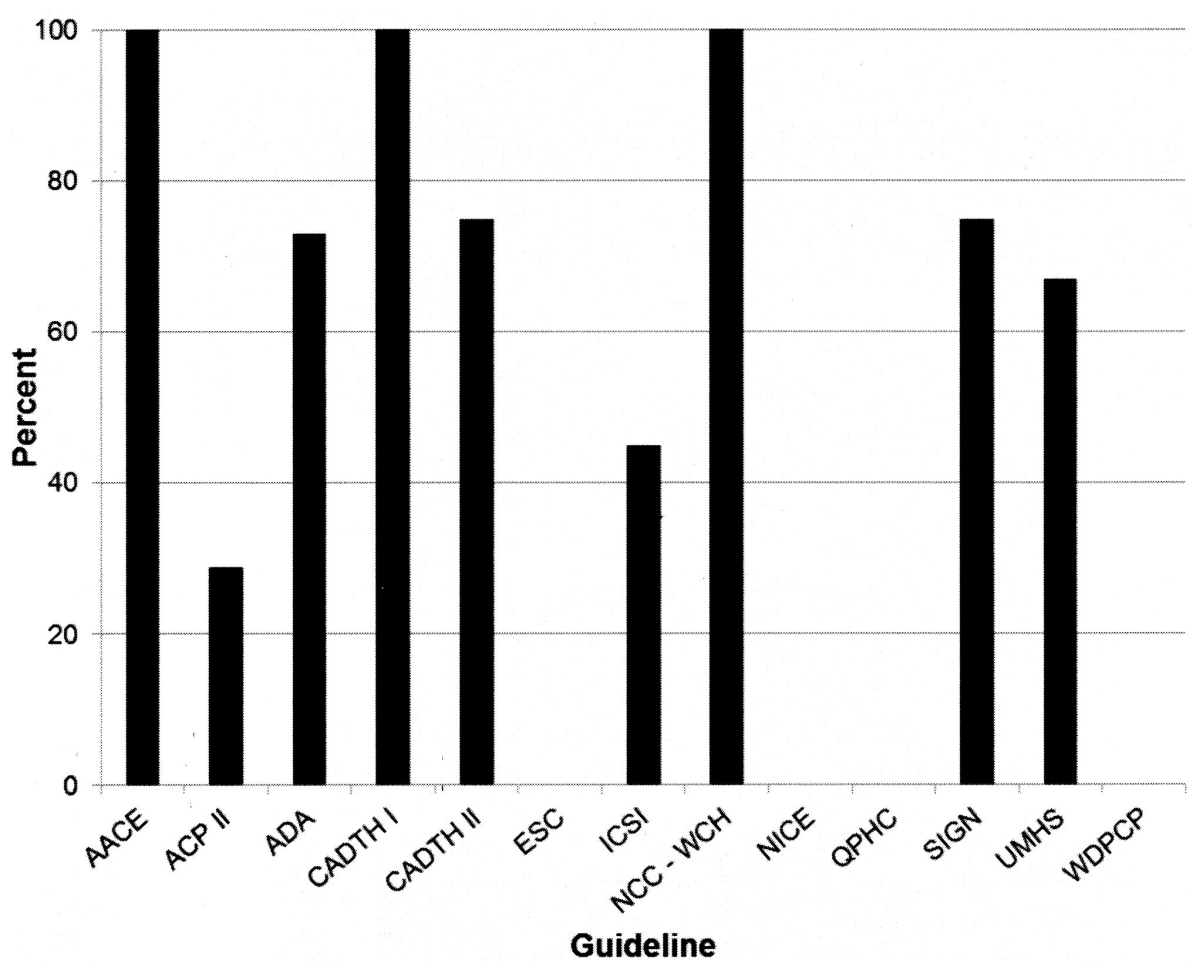
Manufacturers of recommended drugs represented on guideline panels. Percent of manufacturers of patented drugs recommended in each clinical practice guideline for which one or more guideline authors had a financial interest. Abbreviations. See list for Table 1.

On average, 56.1% of manufacturers of patented drugs recommended in each guideline had one or more individuals with a disclosed interest in that company on the guideline panel (median 70.0%, range 0 to 100%) (Figure **3**). Three CPGs had one or more authors with financial interests in all drugs recommended in the guideline [29,31,36]. For example, the AACE [31] guideline recommended drugs made by five different manufacturers, and one or more panel members disclosed financial interests in all five of those companies. 

Of the 12 guidelines with a designated chair, six chairs disclosed one or more financial COI. Four of those six chairs reported relevant interests, with an interest in the manufacturer of 100% (AACE) [31], 45% (ADA) [29], 38% (SIGN) [39], and 33% (CADTH I) [32] recommended drugs.

In the final logistic regression model, none of the COI variables for either the authors or the chair was associated with recommendations for a drug. Among the other CPG characteristics, a US-based CPG was more likely to recommend a specific drug compared to a non-US based CPG (odds ratio [OR] 5.01, 95% confidence interval [CI] 1.80 to 14.0; *P*-value = 0.002). The number of authors was negatively associated with favorably recommending a drug with an OR of 0.84 (95% CI 0.76 to 0.93; *P*-value = 0.001) for each additional author. Other CPG characteristics were not associated with a recommendation to use a specific drug. 

 Overall, there was excellent agreement between the two coauthors on the assessments of whether a drug was recommended in each guideline with a kappa statistic of 0.86 (range 0.40 to 1.00 across the different drugs) and 92.8% agreement (range 81.8% to 100%). 

## Discussion

The chair and other authors of a significant percentage of CPGs on glycemic control in type 2 diabetes disclosed financial interests related to specific patented drugs recommended in those guidelines. On the other hand, individuals with no disclosed financial COI authored three guidelines, and no relevant financial interests were reported in a fourth guideline. We found, however, no statistically significant relationship between drugs recommended in guidelines and the disclosed interests of panel members. 

 This work adds to the existing evidence suggesting that financial COI is highly prevalent among authors of CPGs [19-26]. We report a prevalence of COI among authors of diabetes guidelines that is similar to the rate noted by Neuman and colleagues [26]: 48% of panel members producing CPGs on the management of diabetes or hyperlipidemia disclosed a COI. Our work examined a larger and international cohort of diabetes guidelines, and we explored the relationship between recommendations for specific drugs in each guideline and the financial interests of the guideline authors – work that has not, to our knowledge, previously been published. 

 There are important implications of our findings for both users and developers of CPGs for diabetes specifically, and for CPGs in other clinical areas. Two main concerns arise when guideline authors have financial interests in the topic of the guidelines they are formulating: the risk of bias in the recommendations and the diminished credibility of the CPG. Our study design did not enable us to examine actual bias because it is not possible to definitely declare what is the “right” drug for glycemic control in specific clinical situations: there are invariably reasonable alternatives. However, users of CPGs where the authors have COI need to contemplate the risk of biased recommendations and consider using guidelines where authors do not have such interests. In addition, guideline developers may undermine their own credibility and that of their organization and its products, when they formulate recommendations on drugs with which they have personal financial interests. 

Guideline users should critically appraise any guideline they are considering implementing, using a tool such as AGREE-II [43]. Although imperfect, such an assessment may assist the user in identifying potential sources of bias, such as poorly performed or nonexistent systematic reviews, or lack of transparency in the translation of the body of evidence into recommendations. It is possible that the relevant secondary interests of the authors, if any, may be reflected in one or more of these steps in guideline development. We did not note this relationship, however: the quality of guidelines in our cohort, both those authored by individuals with no relevant COI and those with a high percentage of authors with COI, varied considerably in an assessment using AGREE-II, as reported in elsewhere [44]. 

 In order to produce trustworthy CPGs, the US Institute of Medicine (IOM) [1] recommends that all panel members disclose their financial and other interests; that these interests should be discussed and managed; whenever possible guideline development group members should not have COI; members with COIs should represent not more than a minority of the guideline panel; the chair or co-chairs should not be persons with COI; funders should have no role in CPG development; and the panel members should be multidisciplinary and balanced. It is clear from our study that most guidelines on glycemic control in type 2 diabetes do not meet these standards. 

 It is possible to develop guidelines using persons without relevant interests, as demonstrated by four guidelines in our cohort [34,37,38,41]. Our work also suggests that guideline panels encompassing a larger number of members are less likely to recommend drugs with which the panel has financial interests. This is consistent with the US IOM recommendation that panels have broad representation. 

 In some situations it may be unavoidable that panel members have financial interests in recommended interventions. For CPGs on rare diseases, for example, expertise is likely confined to a small number of individuals who may well have received money from private funders. It is, however, possible to complement those individuals with other relevant scientific and clinical expertise, as well as representation from patients and caregivers. 

Nonfinancial COI may be even more important than financial interests as a source of bias in primary research, systematic reviews, and CPGs [45-49]. In our cohort of guidelines developers, only five requested such disclosures, and no disclosures of nonfinancial interests were made (which does not mean that none were present). Important areas for future research include how to elicit relevant nonfinancial interests from guideline authors, how to report those interests, and most importantly how those interests influence individual and group decision making. 

The relationship between financial interests and recommendations in CPGs is complex and many factors may explain our inability to demonstrate a significant association between recommended drugs and guideline authors’ specific interests. Guideline authors often had financial relationships with a number of different drug manufacturers, and it is unknown how this affects decision-making on specific drugs [50]. It is possible that financial interests in a number of drug manufacturers correlate with recommendations for pharmacotherapy in general, compared to lifestyle interventions; this is, at present, unknown. The relationship between the monetary value of interests and decisions is largely unknown, and most guidelines did not provide specific values for these interests. It is possible that guideline authors’ financial interests, including professional ones, are served by guidelines that focus generally on pharmacotherapy compared to behavioral interventions; author relationships to specific drugs may be less important. It would be interesting to compare disclosures of interests of authors of behavioral intervention guidelines versus the disclosures that we examined in this study. 

The relative timing of payment by drug manufacturers to guideline authors, disclosures of COI, and the formulation of guideline recommendations is variable, and for the most part indeterminate. In addition, drug manufacturers acquire other companies and otherwise evolve their financial interests. Guideline authors change employment: some may have had prior roles with pharmaceutical companies or may be contemplating a new role. It is therefore difficult to examine relationships between disclosed financial interests and specific drug recommendations. 

There are limitations to our study. The examination of recommendations within diabetes guidelines is challenging due to the vast array of available and reasonable treatment options for most populations. Thus the majority of drugs are “recommended” for some patient population in most guidelines, limiting our analysis of the relationship between recommendation of drugs and authors’ financial interests. Our sample size of CPGs was small, providing limited power to assess associations with recommendations.

 Individual panel members disclose interests, however recommendations are made by guideline panels as a whole. It is thus not possible to examine how individual interests relate to individual decisions: we had to assume that all recommendations were made by consensus with ultimate agreement among panel members. We did examine the interests of the chair, however, as we considered their role potentially dominant. 

 In this study we relied on self-report of interests by guideline panel members, as published in guidelines and in the NGC summary. Studies report high rates of nondisclosure or inaccurate disclosure of financial interests by physician authors, however [25,51-54]. Databases that include all financial payments to healthcare providers, as is under development in the US [55], should help to ensure accurate information. 

 The applicability of our findings to other cohorts of CPGs is unclear. We selected type 2 diabetes for this case study because of its high treatment costs and personal burden, and the varied pharmacotherapeutic options. Our findings may apply to other diseases with these characteristics, such as cardio- and cerebrovascular disease prevention and treatment: further research is needed to examine other and larger cohorts of CPGs. In addition, we examined only guidelines with disclosures of COI. Guidelines without disclosures may have an even higher prevalence of authors with relevant COI. It would be interesting to explore the relationship between author financial interests and recommendations in such guidelines. Increased public access to industry payments to physicians [55] makes such research possible. 

 Not only is there a high prevalence of financial interests among the authors of guidelines on pharmacotherapy for glycemic control in type 2 diabetes, but these authors have financial interests in the companies whose patented drugs they are recommending. We did not, however, demonstrate a significant relationship between guideline author disclosures of financial interests and the specific drugs recommended in this small cohort of guidelines. The potential for financial interests to produce actual bias in recommendations needs further research, despite the methodologic challenges. The credibility of these guidelines is diminished by our findings, however, and guideline developers may need to make changes in the composition of guideline development groups in order to publish more trustworthy guidelines in future. 

## Supporting Information

Table S1 **Diabetes drugs and guideline authors' interests" in MS.**
(DOCX)
