# Supplementary material for: Conflicts of Interest among Authors of Clinical Practice Guidelines for Glycemic Control in Type 2 Diabetes Mellitus
Source: PLoS One. 2013 Oct 14;8(10):e75284. doi: 10.1371/journal.pone.0075284 (PMC3796568; doi:10.1371/journal.pone.0075284)
Supplement: Table S1 — Diabetes drugs and guideline authors' interests" in MS. (DOCX) [file pone.0075284.s001.docx]

**Supporting information**

**Table. Diabetes drugs and guideline authors’ interests**

Diabetes drugs and the percentage of authors of each guideline with a disclosed interest in the manufacturer of each drug.

Y/N: Yes or No, the drug was recommended in the guideline.

Abbreviations. See list for Table 1.

| **Drug** | **Manufacturer*** | **AACE** | | **ACP II** | | **ADA** | | **CADTH I** | | **CADTH II** | |
| --- | --- | --- | --- | --- | --- | --- | --- | --- | --- | --- | --- |
|  |  | Drug recommended | % of authors with COI | Drug recommended | % of authors with COI | Drug recommended | % of authors with COI | Drug recommended | % of authors with COI | Drug recommended | % of authors with COI |
| Exenatide | Amylin | N | 22 | Y | 0 | Y | 25 | N | 0 | N | 0 |
| Pramlintide | Amylin | Y | 22 | N | 0 | N | 25 | N | 0 | N | 0 |
| Liraglutide | Novo Nordisk | N | 65 | Y | 0 | Y | 38 | N | 17 | N | 25 |
| Sitagliptin | Merck | N | 52 | Y | 14 | Y | 19 | N | 33 | Y | 33 |
| Saxagliptin | Bristol Myers Squibb | N | 43 | Y | 0 | Y | 13 | N | 0 | Y | 0 |
| Vildagliptin | Novartis | N | 17 | N | 0 | Y | 0 | N | 8 | N | 8 |
| Linagliptin | Boehringer Ingelheim, Eli Lilly | N | 65 | N | 0 | Y | 44 | N | 25 | N | 17 |
| Pioglitazone | Takeda | N | 22 | Y | 0 | Y | 13 | N | 0 | Y | 0 |
| Rosiglitazone | GlaxoSmithKline | N | 52 | Y | 0 | Y | 0 | N | 25 | Y | 25 |
| Repaglinide | Novo Nordisk | N | 65 | Y | 0 | Y | 38 | N | 17 | Y | 25 |
| Nategelinide | Novartis | N | 17 | Y | 0 | Y | 0 | N | 8 | Y | 8 |
| Miglitol | Pfizer | N | 13 | N | 14 | Y | 0 | N | 8 | N | 17 |
| Glimepiride | Sanofi-aventis | N | 26 | N | 7 | N | 31 | N | 17 | N | 33 |
| Colesevelam | Daiichi Sankyo | Y | 17 | N | 0 | Y | 6 | N | 0 | N | 0 |
| Regular human insulin | Eli Lilly, Novo Nordisk | Y | 78 | N | 0 | N | 44 | Y | 42 | Y | 33 |
| Insulin NPH | Eli Lilly, Novo Nordisk | Y | 78 | N | 0 | N | 44 | Y | 42 | Y | 33 |
| Premixed reg/NPH | Eli Lilly, Novo Nordisk | Y | 78 | N | 0 | N | 44 | N | 42 | Y | 33 |
| Insulin lispro | Eli Lilly | Y | 52 | N | 0 | N | 31 | Y | 25 | Y | 17 |
| Insulin aspart | Novo Nordisk | Y | 65 | N | 0 | N | 38 | Y | 17 | Y | 25 |
| Insulin detemir | Novo Nordisk | Y | 65 | N | 0 | N | 38 | Y | 17 | Y | 25 |
| Insulin glargine | Sanofi-aventis | Y | 26 | N | 7 | N | 31 | Y | 17 | Y | 33 |
| insulin glulisine | Sanofi-aventis | Y | 26 | N | 7 | N | 31 | N | 17 | Y | 33 |
| Biphasic insulin lispro | Eli Lilly | Y | 52 | N | 0 | N | 31 | Y | 25 | Y | 17 |
| Biphasic insulin aspart | Novo Nordisk | Y | 65 | N | 0 | N | 38 | Y | 17 | Y | 25 |

| **Drug** | **Manufacturer*** | **ESC**** | | **ICSI** | | **NCC - WCH** | | **NICE** | | **QPHC** | |
| --- | --- | --- | --- | --- | --- | --- | --- | --- | --- | --- | --- |
|  |  | Drug recommended | % of authors with COI | Drug recommended | % of authors with COI | Drug recommended | % of authors with COI | Drug recommended | % of authors with COI | Drug recommended | % of authors with COI |
| Exenatide | Amylin | N | 0 | Y | 15 | N | 0 | N | 0 | N | 0 |
| Pramlintide | Amylin | N | 0 | Y | 15 | N | 0 | N | 0 | N | 0 |
| Liraglutide | Novo Nordisk | N | 11 | Y | 8 | N | 33 | Y | 0 | N | 0 |
| Sitagliptin | Merck | N | 33 | Y | 31 | N | 25 | N | 0 | Y | 0 |
| Saxagliptin | Bristol Myers Squibb | N | 0 | Y | 0 | N | 8 | N | 0 | N | 0 |
| Vildagliptin | Novartis | N | 15 | N | 0 | N | 17 | N | 0 | N | 0 |
| Linagliptin | Boehringer Ingelheim, Eli Lilly | N | 15 | N | 8 | N | 17 | N | 0 | N | 0 |
| Pioglitazone | Takeda | N | 11 | Y | 0 | N | 25 | N | 0 | Y | 0 |
| Rosiglitazone | GlaxoSmithKline | N | 4 | Y | 0 | N | 33 | N | 0 | Y | 0 |
| Repaglinide | Novo Nordisk | N | 11 | Y | 8 | N | 33 | N | 0 | Y | 0 |
| Nategelinide | Novartis | N | 15 | Y | 0 | N | 17 | N | 0 | Y | 0 |
| Miglitol | Pfizer | N | 26 | Y | 8 | N | 8 | N | 0 | N | 0 |
| Glimepiride | Sanofi-aventis | N | 11 | N | 15 | N | 17 | N | 0 | Y | 0 |
| Colesevelam | Daiichi Sankyo | N | 11 | Y | 0 | N | 0 | N | 0 | N | 0 |
| Regular human insulin | Eli Lilly, Novo Nordisk | N | 15 | Y | 8 | N | 25 | N | 0 | Y | 0 |
| Insulin NPH | Eli Lilly, Novo Nordisk | N | 15 | Y | 8 | N | 25 | N | 0 | Y | 0 |
| Premixed reg/NPH | Eli Lilly, Novo Nordisk | N | 15 | Y | 8 | N | 25 | N | 0 | Y | 0 |
| Insulin lispro | Eli Lilly | N | 7 | Y | 8 | Y | 17 | N | 0 | Y | 0 |
| Insulin aspart | Novo Nordisk | N | 11 | Y | 8 | Y | 33 | N | 0 | Y | 0 |
| Insulin detemir | Novo Nordisk | N | 11 | Y | 8 | N | 33 | N | 0 | N | 0 |
| Insulin glargine | Sanofi-aventis | N | 11 | Y | 15 | N | 17 | N | 0 | Y | 0 |
| insulin glulisine | Sanofi-aventis | N | 11 | Y | 15 | N | 17 | N | 0 | N | 0 |
| Biphasic insulin lispro | Eli Lilly | N | 7 | Y | 8 | N | 17 | N | 0 | N | 0 |
| Biphasic insulin aspart | Novo Nordisk | N | 11 | Y | 8 | N | 33 | N | 0 | N | 0 |

| ***Drug*** | **Manufacturer*** | **SIGN** | | **UMHS** | | **WDPCP** | |
| --- | --- | --- | --- | --- | --- | --- | --- |
|  |  | Drug recommended | % of authors with COI | Drug recommended | % of authors with COI | Drug recommended | % of authors with COI |
| Exenatide | Amylin | Y | 0 | Y | 0 | Y | 0 |
| Pramlintide | Amylin | N | 0 | Y | 0 | Y | 0 |
| Liraglutide | Novo Nordisk | Y | 55 | N | 13 | Y | 0 |
| Sitagliptin | Merck | Y | 0 | Y | 13 | Y | 0 |
| Saxagliptin | Bristol Myers Squibb | Y | 9 | N | 0 | Y | 0 |
| Vildagliptin | Novartis | Y | 9 | N | 0 | N | 0 |
| Linagliptin | Boehringer Ingelheim, Eli Lilly | N | 18 | N | 25 | Y | 0 |
| Pioglitazone | Takeda | Y | 18 | Y | 13 | Y | 0 |
| Rosiglitazone | GlaxoSmithKline | Y | 55 | Y | 13 | Y | 0 |
| Repaglinide | Novo Nordisk | N | 55 | Y | 13 | Y | 0 |
| Nategelinide | Novartis | N | 9 | Y | 0 | Y | 0 |
| Miglitol | Pfizer | N | 9 | Y | 0 | Y | 0 |
| Glimepiride | Sanofi-aventis | N | 18 | N | 13 | N | 0 |
| Colesevelam | Daiichi Sankyo | N | 9 | N | 0 | N | 0 |
| Regular human insulin | Eli Lilly, Novo Nordisk | Y | 55 | Y | 25 | Y | 0 |
| Insulin NPH | Eli Lilly, Novo Nordisk | Y | 55 | Y | 25 | Y | 0 |
| Premixed reg/NPH | Eli Lilly, Novo Nordisk | N | 55 | Y | 25 | Y | 0 |
| Insulin lispro | Eli Lilly | Y | 18 | Y | 25 | Y | 0 |
| Insulin aspart | Novo Nordisk | Y | 55 | Y | 13 | Y | 0 |
| Insulin detemir | Novo Nordisk | N | 55 | Y | 13 | Y | 0 |
| Insulin glargine | Sanofi-aventis | N | 18 | Y | 13 | Y | 0 |
| insulin glulisine | Sanofi-aventis | N | 18 | Y | 13 | Y | 0 |
| Biphasic insulin lispro | Eli Lilly | N | 18 | Y | 25 | Y | 0 |
| Biphasic insulin aspart | Novo Nordisk | N | 55 | Y | 13 | Y | 0 |
